# Pine Sawmill Waste-Derived Graphene Derivatives for Cementitious Composites

**DOI:** 10.1021/acsaenm.6c00203

**Published:** 2026-05-18

**Authors:** Iftekhar Alam Dipta, Anthony R. Richard, Jacob Heil, Chooi Kim Lau, Kam Ng

**Affiliations:** † Department of Civil and Architectural Engineering and Construction Management, 4416 University of Wyoming, 1000 E. University Avenue, Laramie, Wyoming 82071, United States; ‡ Acadian Research & Development LLC, 1482 Commerce Dr. Unit X, Laramie, Wyoming 82070, United States

**Keywords:** graphene oxide, reduced graphene oxide, biochar-based graphene, pyrolysis temperature, cementitious composites

## Abstract

Graphene oxide (GO) and reduced graphene oxide (rGO), synthesized from pinewood waste biomass through a pyrolysis-temperature-controlled route at 450, 600, and 900 °C, were investigated for their influence on the hydration behavior, microstructure, and mechanical performance of cementitious composites at both cement paste and concrete scales. The graphene materials synthesized from biochar in this work are designed as replacements for conventionally synthesized GO and/or rGO from graphite, offering comparable performance while enabling production from renewable sources at an accessible cost. Comprehensive material characterization confirmed the successful formation of partially layered carbon structures. Incorporation of GO and rGO, which are synthesized from biochar by referring to BCGO and BCrGO, respectively, into cement pastes at a fixed dosage (0.05% by weight of cement (BWOC), used as an additive) resulted in accelerated hydration kinetics and enhanced development of hydration products without the formation of additional crystalline phases, indicating refinement of the hydration process rather than alteration of cement chemistry. Microstructural analysis revealed strengthened Si–O–Si and Si–O–Ca bonding environments, accompanied by a denser and more homogeneous cementitious system. Microphotographic observations further confirmed improved interfacial bonding between hydration products and graphene materials. At the concrete scale, the addition of GO and rGO at varying dosages (0.05 and 0.5% BWOC) as concrete additives resulted in consistent improvements in mechanical performance. Compressive strength increased by up to 10%, while flexural and tensile strengths improved by about 9% from all mixes. The most pronounced property enhancement was observed in the modulus of elasticity, with up to 55% enhancement. Concrete density, pH, and Poisson’s ratio remained essentially unchanged, indicating improved mechanical properties without compromising ductility or chemical stability. These findings highlight the potential of renewable biochar-based GO and rGO as high-performance additives at the desired pyrolysis temperature, capable of enhancing mechanical and microstructural performance without compromising essential cementitious properties.

## Graphical abstract

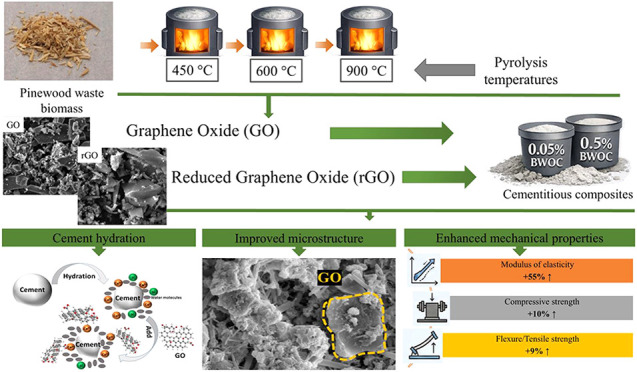

## 1 Introduction

Concrete remains the most widely used construction material in the world, yet it is also one of the largest contributors to anthropogenic carbon emissions. The global cement industry accounts for nearly 8% of total CO_2_ emissions, largely due to the high clinker content and energy-intensive calcination processes required for cement production.^1–3^ As modern infrastructure demands continue to rise, improving the efficiency and performance of concrete is a direct pathway for reducing its embodied carbon footprint.^4,5^ Among emerging strategies, the incorporation of carbon-based materials,^6–8^ particularly graphene oxide (GO)^9–13^ and its derivative form, and reduced graphene oxide (rGO),^14–18^ has attracted significant attention due to their ability to enhance hydration kinetics, refine microstructure, and improve mechanical performance at very low dosages. GO consists of two-dimensional carbon nanosheets decorated with oxygen-containing functional groups, such as hydroxyl, epoxy, and carboxyl groups, which promote dispersion in aqueous media and facilitate interactions with calcium and silicate ions during early hydration.^10^ These functional groups act as nucleation sites for calcium silicate hydrate (CSH) formation, accelerating hydration reactions and contributing to microstructural refinement.^11^ In contrast, rGO, obtained by partial removal of oxygen functionalities, exhibits a higher degree of sp^2^ *-hybridized* carbon (sp^2^) restoration, resulting in improved stiffness and structural continuity but reduced chemical reactivity.^15^ Evaluating both materials enables identification of the balance between chemical reactivity and mechanical reinforcement governing cementitious performance. Studies have reported that incorporating graphene derivatives, both GO and rGO, at very low dosages (≤0.5% BWOC) can significantly improve the performance of cementitious materials.^12,18^ At lower dosages, GO contents in the range of 0.03–0.05% BWOC have been shown to enhance concrete compressive strength by approximately 12–33% and flexural strength by 41–59%.^11,19–21^ Similar or greater efficiency has been reported for rGO, where additions below 0.1% BWOC led to compressive strength increases of approximately 20–30% both in cement paste and concrete, with comparative studies indicating superior performance of rGO over GO at identical dosages (∼0.1% BWOC).^14–18,22^ These macroscopic improvements are closely associated with hydration-related mechanisms, as GO or rGO dosage, dispersion state, nanosheet size, and oxygen-containing functional groups strongly influence hydration kinetics and microstructural development.^15^ An increase in the content of graphene derivatives typically accelerates cement hydration and increases the degree of hydration.^21^ At low dosages, thinner, smaller nanosheets facilitate the formation of flower-like hydration products, resulting in a dense, compact paste.^19^

Despite their demonstrated performance benefits, the widespread adoption of GO as a concrete additive remains limited by cost and scalability.^19,20^ The economics of GO production pose a significant barrier to large-scale industrial integration, with commercial prices ranging from $1000 to over $60,000 per kilogram for common graphene derivatives, depending on quality and purity.^23–26^ This results in price increases that dwarf the cost benefits related to the observed performance boost when used in concrete. Addressing this issue by producing similar performance-enhancing materials at more accessible prices for general use is the primary motivation for this work. Most GO used in research and commercial applications is derived from graphite^14^ via the Brodie method,^27^ Hummers,^28^ or modified Hummers oxidation routes,^29^ which rely on concentrated acids and strong oxidizing agents such as KMnO_4_ and H_2_SO_4_.^30^ Graphite is an expensive, nonrenewable resource with limited domestic availability in many regions, rendering such GO economically impractical for large-volume construction materials [18]. These constraints have prompted growing interest in renewable, low-cost biochar-based precursors for the synthesis of graphene oxide and its derivatives.^31^ In this study, wood-derived biochar is selected as the precursor for the synthesis of GO and rGO, providing an abundant, renewable, and carbon-rich alternative to conventional graphite sources. When pyrolyzed under limited oxygen, wood biomass forms a stable carbonaceous structure with aromatic domains conducive to oxidation and subsequent functionalization. Despite growing interest in wood-derived biochar-based (WB) materials, there remains a limited understanding of how biochar precursor characteristics, particularly those influenced by pyrolysis temperature, affect the structures of biochar-based GO (BCGO) and rGO (BCrGO), and how these structures affect cement hydration and concrete performance. It has been found that altering pyrolysis conditions results in biochars with very different properties,^32^ which in turn influences the properties (especially oxygen content) of the resulting BCGO and BCrGO products and drives the necessity for investigation into the effect of precursor processing methods on the final products’ performance in concrete. This knowledge gap has largely constrained the rational design of BDGM for construction applications.

The synthesis approach adopted in this study differs fundamentally from conventional graphite-derived GO and rGO methods reported in the literature. Traditional routes typically involve multiple processing steps and the use of concentrated acids and strong oxidizing agents, raising concerns regarding safety, environmental impact, and scalability.^27,33,34^ In particular, conventional Hummers-based synthesis generates large volumes of acidic waste and manganese-containing residues, which require extensive neutralization and disposal.^35^ In contrast, the method applied herein utilizes a single-reagent nitric acid route at relatively low temperature, enabling a simpler, faster, and more environmentally compatible synthesis process.^36^ This wood-derived pathway eliminates dependence on mineral graphite and can be readily integrated with biochar production from forestry or agricultural waste streams while also allowing for extensive recycling of reactants. Such an approach aligns with circular economy principles, in which carbon is recycled from biomass into long-lasting construction materials rather than emitted as CO_2_. Additionally, the simplified processing and recyclability of reagents significantly improve the overall economic viability of the materials.

In this newly developed pyrolysis process, biochar is exfoliated via sonication, and nitric acid introduces oxygen-containing functional groups, producing BCGO,^36^ which is subsequently thermally reduced to obtain BCrGO. During biochar production, three pyrolysis temperatures, 450, 600, and 900 °C, were selected from an initial set of candidate temperatures to represent low-, intermediate-, and high-temperature regimes. These temperatures produce precursor materials with substantially different physicochemical properties, including variations in oxygen content and structural ordering. These differences were confirmed through material characterization techniques. In turn, these variations influence the properties of the resulting GO and rGO materials, which may affect their performance in concrete. The resulting WB obtained at all pyrolysis temperatures does not share the single- to few-layer morphology typical of graphite-derived GO/rGO and possesses a more amorphous structure; however, it does contain regions of sp^2^-hybridized carbon and remains conducive to oxidation.^31^ Despite this morphological distinction, the materials retain sufficient functional-group density and nanoscale dispersion to activate hydration and reinforcement mechanisms comparable to those of graphite-derived GO and/or rGO. These characteristics enable the WB to offer comparable benefits when incorporated into concrete as graphite-derived GO and/or rGO.^12^ Most previous studies have not explicitly considered this thermal-history variable, despite its strong influence on nanocarbon surface chemistry and performance. A quantitative comparison between conventional graphite-derived graphene materials and the BCGO and BCrGO developed in this study is presented in Table 1, highlighting key differences in synthesis routes, carbon-to-oxygen ratios, and economic feasibility. This study systematically links pyrolysis-temperature-controlled WB to hydration kinetics, microstructural evolution, and mechanical performance of cement paste and concrete at low graphene dosages relevant to practical construction.

**Table 1:** Comparative Physicochemical and Economic Characteristics of Graphite-Derived and Biochar-Derived Graphene Oxide (GO) and Reduced Graphene Oxide (rGO)

**property**	**graphite-derived GO**	**BCGO (this study)**	**graphite-derived rGO**	**BCrGO (this study)**
**synthesis route**	hummers or variation of hummers	HNO_3_, water, sonication	thermal reduction (1000 °C)	thermal reduction (1100 °C)
**carbon/oxygen (C/O) ratio**	1.98–2.9 ^37^	450 °C: 3.4	6.2–14.7 ^38^	>20 for all precursor pyrolysis temperatures
		600 °C: 4.0		
		900 °C: 5.3		
**cost**	$1000–$28,500/kg^25,26^	<$30/kg	$1000–$62,450/kg^23,24^	<$50/kg

The findings of this study aim to address two interconnected objectives: (1) advancing the understanding of structure–property relationships in WBs and (2) evaluating the feasibility of these newly developed materials for large-scale application in the construction industry. By linking material synthesis with multiscale characterization of cementitious systems and mechanical performance evaluation, this work contributes to the growing effort to capture and retain carbon in construction materials over extended service lifetimes, while opening new opportunities for valorizing biomass waste streams into high-value graphene-based additives.

## 2 Raw Materials

### 2.1 Biochar, BCGO, and BCrGO

Feedstock pinewood, derived as waste from a commercial pine sawmill, was used as the precursor material. Biochar (BC) was produced from that pine feedstock under a flowing nitrogen atmosphere at target pyrolysis temperatures of 450, 600, and 900 °C using an electric tube furnace. The heating rate was maintained at 10 °C/min, and the final temperature was held for 2 h to ensure complete carbonization. The resulting biochar was subsequently ground in a planetary ball mill and sieved to a particle size below 43 μm. The milled biochar was then dispersed in diluted nitric acid and subjected to sonication at 70 °C for 4 h to synthesize BCGO via a method developed at the University of Wyoming, WY, USA.^36^ After completion of the reaction, the mixture was quenched with deionized water, thoroughly filtered and washed, and then dried to form powder BCGO, yielding a final pH of approximately 3.

### 2.2 Cement

In this research, ordinary Portland cement (OPC) conforming to ASTM C150/C150 M^39^ specification for Type I/II was used in all cementitious mixes. The specific surface area of this cement was found to be 0.903 m^2^/g^40^ with a specific gravity of 3.15. The oxide composition showed that the cement was primarily composed of CaO (63.33%) and SiO_2_ (19.66%), along with Al_2_O_3_ (4.36%), Fe_2_O_3_ (3.41%), and SO_3_ (3.62%), with minor amounts of MgO (1.22%), K_2_O (0.81%), Na_2_O (0.13%), and other trace oxides. In addition, phase analysis confirmed that the primary clinker phases consisted of 63.90% tricalcium silicate (C_3_S), 8.16% dicalcium silicate (C_2_S), 5.79% tricalcium aluminate (C_3_A), and 10.37% tetracalcium aluminoferrite (C_4_AF), along with minor crystalline phases including gypsum dihydrate (CaSO_4_·2H_2_O), gypsum hemihydrate (CaSO_4_·0.5H_2_O), and portlandite (Ca­(OH)_2_).

### 2.3 Aggregates

Crushed granite with a maximum particle size of 12.5 mm was used as coarse aggregate (CA). The CA had a dry-rodded unit weight of 1590.73 kg/m^3^,^41^ a water absorption of 0.16%,^42^ a moisture content of 1.0%,^43^ a fineness modulus of 7.78,^44^ and bulk and apparent specific gravities in saturated surface dry (SSD) condition of 2.71 and 2.72, respectively.^42^

Natural river sand was used as fine aggregate (FA), with particle sizes ranging from 0.15 to 4.75 mm in accordance with ASTM C33.^45^ The FA had bulk specific gravities of 2.59 (oven-dry) and 2.57 (SSD),^42^ a dry-rodded unit weight of 1586.68 kg/m^3^,^41^ a water absorption of 0.60%,^42^ a moisture content of 3.20%, and a fineness modulus of 2.58.^44^

## 3 Design Mix of Cement Paste and Concrete Samples

Wood-derived biochar-based (WB) materials, including BCGO and BCrGO, were used as additives in the cement paste and concrete. Building on material characterization, paste-level hydration, and microstructural analyses, the investigation was extended to a concrete scale to evaluate physical, mechanical, and elastic properties under varying WB dosages and pyrolysis temperatures.

Cementitious paste mixtures were prepared using cement, water, and WB at a fixed water-to-cement ratio of 0.6, with BCGO and BCrGO incorporated as an additive at 0.05% BWOC. A control mixture was included for each temperature series of 450, 600, and 900 °C. Paste samples were designated using a consistent naming convention (e.g., BCGO0 for the control mix and BCGO450 or BCrGO450 for GO- or rGO-based samples synthesized at 450 °C), which was applied throughout all cement paste analyses.

For concrete, a total of 11 mixtures were prepared (Table 2), incorporating BCGO and BCrGO at dosages of 0, 0.05, and 0.50% BWOC as an additive to concrete. A consistent nomenclature was adopted, in which E denotes concrete mixtures (to distinguish from cement paste), BC refers to biochar-based material, followed by the material type (GO or rGO), synthesis temperature, and dosage level (e.g., EBCGO450–5 or EBCrGO600–50); EBCGO0 represents the control mix. All mixtures were produced at a constant water-to-cement ratio of 0.60 with a fixed mix proportion of 1.0:2.3:2.0 (cement/fine aggregate/coarse aggregate) by weight.

**Table 2:** Mix Design for 1 m^3^ of Concrete (by Weight) Incorporating WB as an Additive to Concrete^a^

**designation**	**% of WB**	**w/c**	**water (kg)**	**cement (kg)**	**WB (kg)**	**FA (kg)**	**CA (kg)**	**total weight (kg)**
EBCGO0	0	0.60	228.00	380.00	0.00	876.00	788.00	2272.00
EBCGO450–5	0.05	0.60	228.00	380.00	0.19	876.00	788.00	2272.19
EBCGO450–50	0.50	0.60	228.00	380.00	1.90	876.00	788.00	2273.90
EBCGO600–5	0.05	0.60	228.00	380.00	0.19	876.00	788.00	2272.19
EBCGO600–50	0.50	0.60	228.00	380.00	1.90	876.00	788.00	2273.90
EBCGO900–5	0.05	0.60	228.00	380.00	0.19	876.00	788.00	2272.19
EBCGO900–50	0.50	0.60	228.00	380.00	1.90	876.00	788.00	2273.90
EBCrGO600–5	0.05	0.60	228.00	380.00	0.19	876.00	788.00	2272.19
EBCrGO600–50	0.50	0.60	228.00	380.00	1.90	876.00	788.00	2273.90
EBCrGO900–5	0.05	0.60	228.00	380.00	0.19	876.00	788.00	2272.19
EBCrGO900–50	0.50	0.60	228.00	380.00	1.90	876.00	788.00	2273.90

a w/c - water-to-cement ratio, WB - specified pyrolysis temperature of wood-derived biochar-based graphene oxide and reduced graphene oxide, FA - fine aggregate, and CA - coarse aggregate.

## 4 Fabrication Process and Testing

The fabrication and testing processes for WB material characterization, cementitious microstructural evaluation, and concrete property assessment were carried out in accordance with standardized procedures to ensure a consistent, accurate, and repeatable. A comprehensive workflow outlining the material preparation, sample casting, and testing stages is presented in Figure 1.

**Figure 1:**
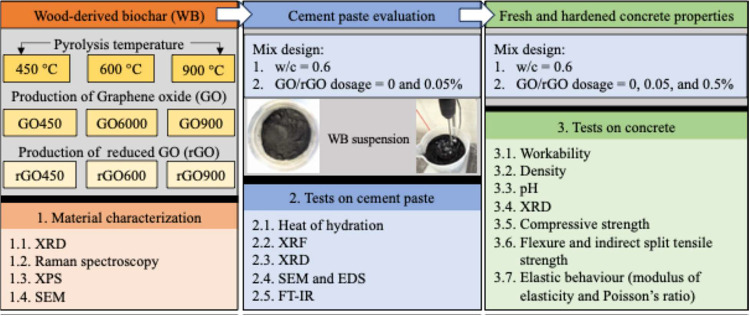
Experimental workflow for WB-incorporated cementitious mix.

### 4.1 BC, GO, and rGO Characterization

The wood-derived biochar-based (WB) materials investigated in this study, including biochar (BC), biochar-based graphene oxide (BCGO), and biochar-based reduced graphene oxide (BCrGO), were characterized by evaluating their structural, thermal, and surface properties. The crystalline structure was examined by X-ray diffraction (XRD) using a Bruker D2 Phaser diffractometer. Raman spectroscopy was used to aid in the determination of bonding and defect characteristics. Additionally, X-ray photoelectron spectroscopy (XPS) was used to examine bonding characteristics, and scanning electron microscopy (SEM) was employed to examine the morphology and surface texture of the WB samples. All characterizations were performed on samples prepared from the same batch of pine sawmill waste, and measurements were conducted on multiple samples to ensure the consistency and repeatability of the results.

### 4.2 Cementitious Paste Preparation and Associated Tests

To evaluate the early age hydration behavior of cement paste incorporating BCGO and BCrGO synthesized at different pyrolysis temperatures (450, 600, and 900 °C), isothermal calorimetry tests were conducted. Paste mixtures were prepared at a constant water-to-cement ratio of 0.6, with BCGO or BCrGO added at 0.05% BWOC, including a control mix. Approximately 75 g of paste was placed in each calorimeter sample cup, and measurements were performed using an I-Cal 8000 HPC calorimeter in accordance with the ASTM C1702 ^46^ at a constant temperature of 23 °C for a duration of 72 h. A single representative sample per mixture was used to evaluate comparative hydration trends under controlled conditions, with all samples prepared from the same cement batch and identical mixing procedures to minimize variability. Separate cement paste batches were prepared for subsequent paste-level characterization tests using the same mix proportions. After casting, the specimens were kept in a sealed environment for 24 h, then demolded, and cured in a wet room at 25 °C and approximately 95% relative humidity until the designated test ages. Changes in chemical composition were evaluated using X-ray fluorescence (Bruker hand-held XRF spectrometer) at 7 and 28 days. At both ages, measurements were conducted directly on the specimen surface with this nondestructive hand-held XRF analyzer without interrupting hydration; therefore, no hydration-stopping procedure was applied. At 28 days, after the XRF measurement, specimens were subsequently crushed and prepared for further microstructural characterization. The crystalline phases of hydrated pastes were examined by XRD using Cu–Kβ radiation over a 2θ (degree) range of 5 to 60°. Microstructural features and elemental distribution were analyzed using field-emission scanning electron microscopy coupled with energy-dispersive spectroscopy (FESEM-EDS) after platinum coating. Fourier-transform infrared (FT-IR) spectroscopy was conducted to assess functional-group evolution, with emphasis on BCGO samples synthesized at 450, 600, and 900 °C and BCrGO synthesized only at 900 °C due to fewer infrared-active functional groups; rGO characterization was therefore supported by other complementary techniques.

### 4.3 Concrete Mixing and Sample Preparation

Concrete mixtures were prepared following the American Concrete Institute (ACI) mix design method.^47^ Coarse and fine aggregates were oven-dried for at least 24 h prior to mixing, following the ASTM C192,^48^ and all materials were batched by weight based on the proportions listed in Table 2. For mixtures incorporating BCGO or BCrGO, the required amount of WB powder was first dispersed in mixing water by using an overhead mechanical stirrer (2000 rpm for 15 min) to obtain a uniform suspension, which was then added to the concrete mixer. Mixing was carried out in a laboratory concrete mixer (0.11 m^3^ capacity) by first combining coarse aggregate with approximately 15% of the total mixing water for 2 min, followed by the addition of fine aggregate, cement, and the remaining water containing the WB suspension. Mixing continued for an additional 4 min, until a uniform consistency was achieved. Fresh concrete workability was immediately evaluated using the slump test in accordance with ASTM C143.^49^

Fresh concrete was then cast into 50 mm × 100 mm cylindrical and 100 mm × 100 mm × 355 mm beam molds in two layers and compacted by rodding in accordance with the ASTM C192.^48^ Cylindrical specimens were rodded 25 times per layer using a 9.5 mm diameter tamping rod, while beam specimens were compacted using a 16 mm diameter rod at a rate of one rodding per 14 cm^2^ of surface area. After casting, specimens were sealed and stored at room temperature for 24 h, followed by curing in a wet room at 25 °C and approximately 95% relative humidity until the designated testing ages.

### 4.4 Concrete Test

For all hardened mechanical and elastic property tests, a minimum of three replicate specimens per mix were tested to ensure accuracy, repeatability, and statistical reliability of the results. Before mechanical testing, the mass and dimensions of each concrete specimen were measured to determine density. Cylindrical specimens were inspected for dimensional accuracy, and end surfaces were ground when necessary to ensure planarity and perpendicular alignment. Uniaxial compressive strength tests were performed on cylindrical specimens at curing ages of 7, 14, 28, and 56 days using an automatic compression testing machine (AC-250MRF, Gilson) in accordance with the ASTM C39,^50^ as shown in Figure 2a. Loading was applied at a constant rate of 0.24 MPa/s, following an initial seating load of 2.22 kN. Flexural strength was determined at 28 days using the four-point bending method in accordance with the ASTM C78 ^51^ on beam specimens, with loading applied at a rate of 1.03 MPa/min [Figure 2b]. Indirect split tensile strength tests were conducted at 28 days on cylindrical specimens following the ASTM C496,^52^ using leather shims and a loading rate of 1.03 MPa/min, as shown in Figure 2c. The modulus of elasticity and Poisson’s ratio were determined at 28 days using a servo-controlled compression system (RTRX-140BX9, GCTS). Axial and radial strains were measured using LVDTs [Figure 2d], and stress–strain data obtained under static loading at an axial strain rate of 0.1% min^–1^ were used to calculate the tangent modulus and Poisson’s ratio from the linear portion of the curve.

**Figure 2:**
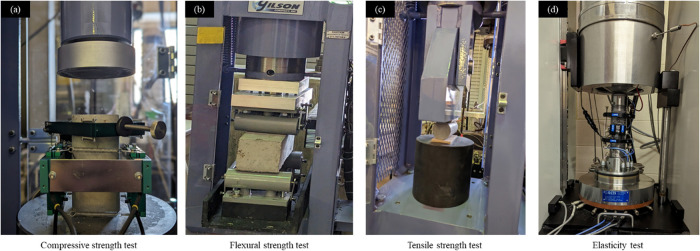
Mechanical tests of concrete.

Post-test microstructural analysis of concrete was conducted using XRD on powdered samples obtained from 28-day-tested compression specimens, then prepared by crushing, sieving to pass through a 75 μm sieve, and oven-drying at 60 °C for 24 h before testing, and scanned from 5 to 60° (2θ) using Cu–Kβ radiation. Concrete pH was measured in accordance with the ASTM D4972-19 ^53^ using powdered samples, passing through a 2 mm sieve, prepared from 28-day-tested compression specimens mixed with distilled water at a 2:1 ratio.

## 5 Results and Discussion

### 5.1 Materials Characterization

#### 5.1.1 X-ray Diffraction (XRD) Analysis

XRD was performed to examine the structural evolution and crystallinity of the WB, as shown in Figure 3a–c. Biochar produced at 450 °C exhibits a broad amorphous hump centered at 21–22° (2θ), indicating predominantly disordered carbon with limited graphitic domains. A weak (002) reflection near 25° (2θ) and diffuse (100) and (101) bands between 42 and 45° (2θ) suggest early formation of carbon layers with significant stacking disorder. With increasing pyrolysis temperature to 600 °C, the broad amorphous peak at 21.8° (2θ) becomes more defined, corresponding to a nongraphitic carbon phase with a *d*-spacing of 0.41 nm as determined by Bragg’s Law. In contrast, a small (002) peak at 25.9° (2θ), corresponding to graphitic carbon, indicates partial graphitic ordering with an interlayer spacing of 0.34 nm and is not associated with any crystalline mineral phase. The broad (100) and (101) reflections at 42.5° and 45.0° (2θ) are characteristic of stacking disorder in carbon structures.^54–56^ Conversion to BCGO results in peak broadening and reduced intensity, reflecting increased structural disorder due to oxidation. This effect is more pronounced in lower-temperature biochar, where the structure of the BC precursor is less ordered. The oxidation process is more effective for lower-temperature BC materials, as reflected in the diffraction patterns. Upon reduction, BCrGO exhibits an increased and slightly shifted disordered peak near 22° (2θ), consistent with oxygen removal and partial contraction of the carbon layers. At 900 °C, a sharper (002) reflection with reduced interlayer spacing is observed, indicating enhanced graphitic stacking and improved structural ordering.

**Figure 3:**
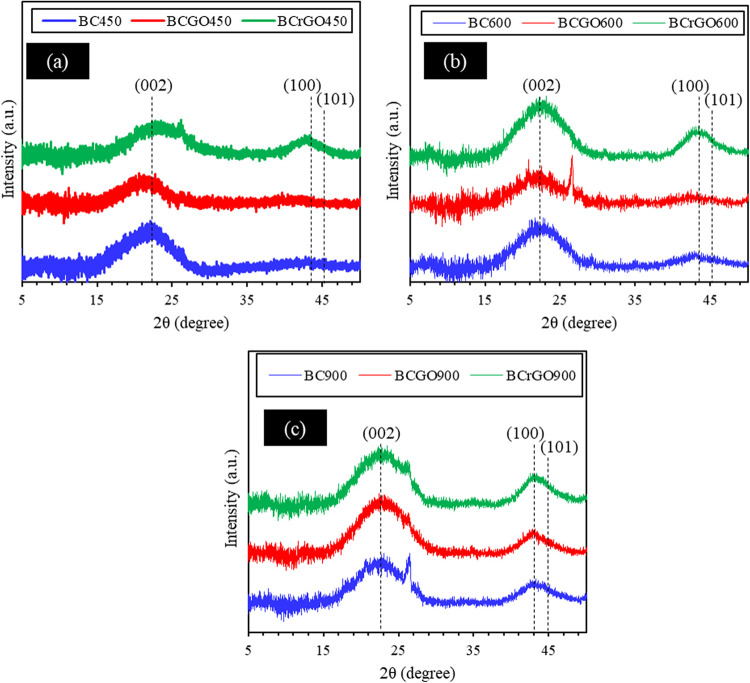
X-ray powder diffraction patterns. (a) BC450, BCGO450, and BCrGO450; (b) BC600, BCGO600, and BCrGO600; (c) BC900, BCGO900, and BCrGO900.

#### 5.1.2 Raman Spectroscopy Analysis

Raman spectroscopy was used to determine structural properties of the WB materials, as it is a useful tool for characterizing a wide variety of carbon species. Two peaks, the D peak at ∼1340 cm^–1^ and the G peak at ∼1585 cm^–1^, are the primary focus for analysis of graphitic carbons. The D peak is related to the breathing mode of carbon rings and is indicative of defects within graphitic materials, and the G peak is associated with sp^2^ hybridized carbon.^57,58^ Peak fitting is frequently employed to identify secondary bands such as the D1 peak, which is located near 1500 cm^–1^ and has been attributed to amorphous carbon.^59^ Curve fitting is also useful for obtaining D and G peak intensities, which are often presented as the D/G ratio which can assist in the determination of structural characteristics such as defect concentration (which can be related to oxidation) and extent of graphitization.^60^ Two pseudo-Voigt peaks were used to fit the D and G peaks, and a Gaussian peak was used to fit the D1 peak.

Figure 4 shows the full-scale Raman spectra for all WB samples, with corresponding D/G peak ratios presented in Table 3. The expected D and G peaks are present in all materials, indicating the presence of defective carbon structures and graphitic domains. When comparing the D/G ratios between biochar, GO, and rGO, the materials produced from 450, 600, and 900 °C biochar follow a similar trend, with D/G ratios increasing from biochar to GO before increasing again from GO to rGO. These increasing trends make sense, as an increase in the D peak can be an indication of structural ordering where amorphous carbon is present, owing to the fact that it is proportional to the presence of six-membered carbon rings in the material. As such, an increase in the D/G ratio upon processing to GO may be related to increased oxygen content and structural changes, while the further increase upon reduction is likely a result of further ordering of amorphous carbon into six-membered ring structures. This is also supported by the narrowing of the D peak width, as widening of the D peak is related to the presence of rings other than the six-membered variety.^58^ The results also show that the D/G ratio increases as charring temperature increases for these materials, indicating that the intensity of thermal treatment influences the structure with higher temperatures inducing the formation of ringed carbon structures. Additionally, there is significantly less change in the D/G ratio for the 900 °C materials, likely owing to the wood having been charred at a temperature closer to that used for reduction, leaving less potential for further changes due to thermal effects.

**Figure 4:**
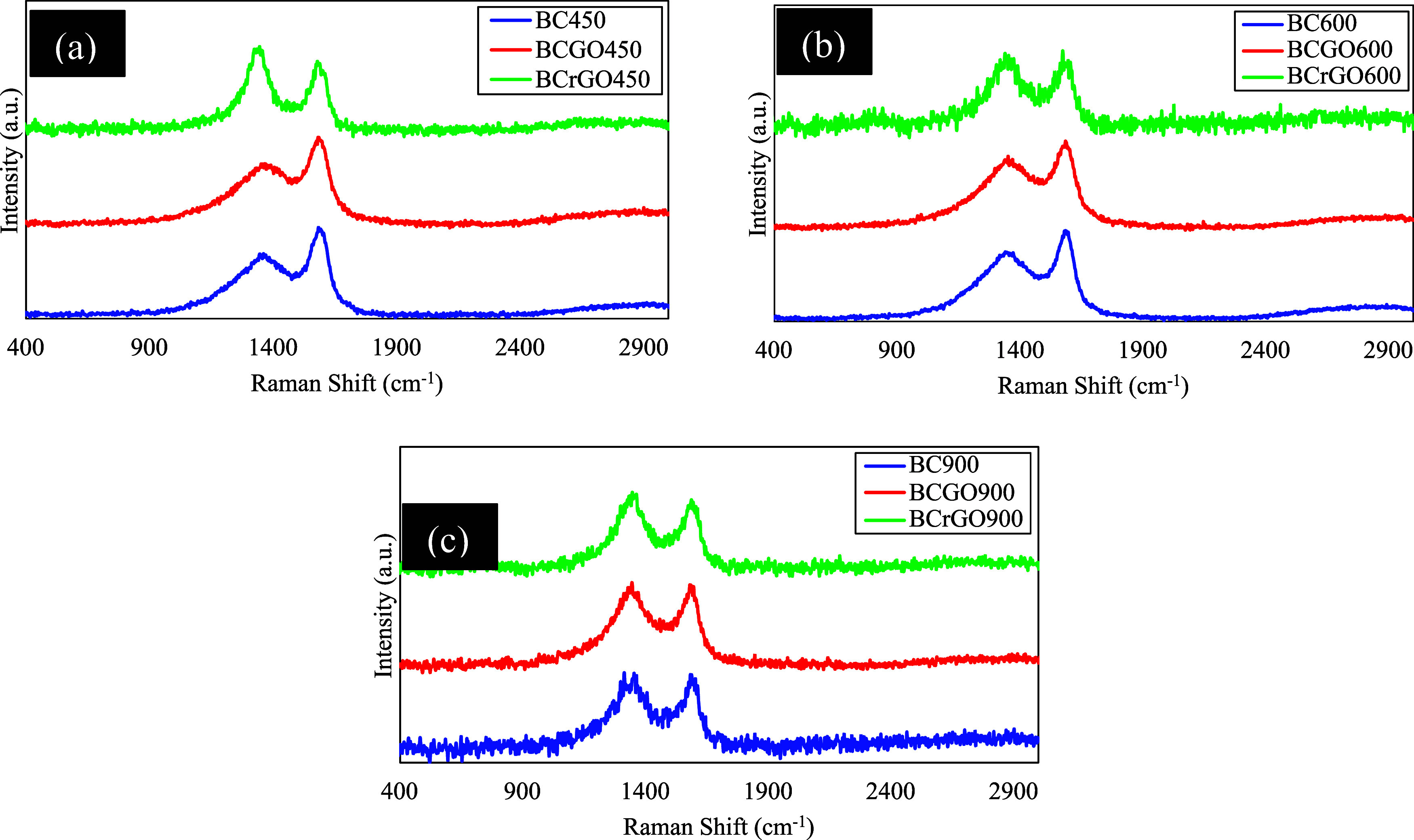
Normalized Raman spectra for all WB materials. (a) BC450, BCGO450, and BCrGO450; (b) BC600, BCGO600, and BCrGO600; (c) BC900, BCGO900, and BCrGO900.

**Table 3:** D/G Peak Ratios for all WB Materials

	**biochar**	**GO**	**rGO**
**temperature (°C)**	**D/G**	**D/G**	**D/G**
450	0.78	0.89	1.25
600	0.84	1.01	1.38
900	1.13	1.16	1.24

#### 5.1.3 X-ray Photoelectron Spectroscopy (XPS) Analysis

XPS was used to evaluate the surface chemistry of the WB materials, with the carbon-to-oxygen (C/O) ratio shown in Figure 5. The C/O ratio was calculated from the relative atomic concentrations of the C 1s and O 1s peaks. BC exhibits moderate C/O ratios (6–8), while BCGO shows substantially lower values (3–5) due to the introduction of oxygen-containing functional groups during oxidation, confirming the successful BCGO synthesis. For both biochar and BCGO, the C/O ratio increases slightly with an increase in pyrolysis temperature. In contrast, BCrGO shows a pronounced increase in C/O ratios (21–27), indicating effective oxygen removal during reduction and the formation of a highly carbon-rich structure.

**Figure 5:**
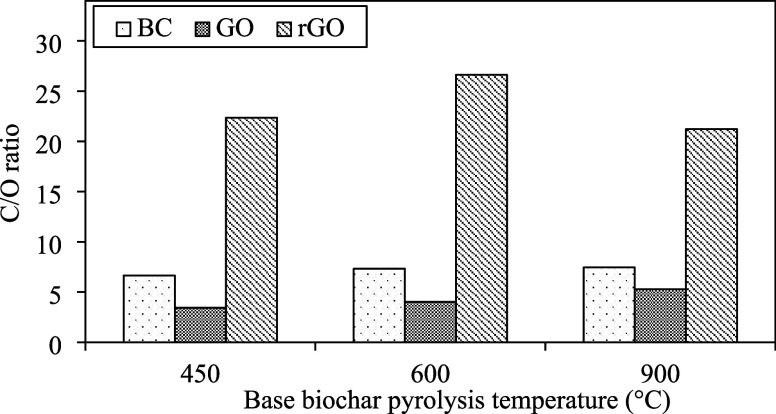
Carbon-to-oxygen (C/O) ratio for WB materials.

#### 5.1.4 Scanning Electron Microscopy (SEM) Analysis

The morphology of the WB with different pyrolysis temperatures was examined by SEM at a 10 μm scale in backscattered electron mode (Figure 6). BC (Figure 6a–c) samples exhibit irregular, fractured particles, with surface smoothness increasing with pyrolysis temperature. BCGO samples (Figure 6d–f) show rougher, etched surfaces due to oxidation, which provide abundant surface irregularities and active sites that favor physical anchoring of hydration products, thereby promoting improved bonding with calcium silicate hydrate (CSH) and other cementitious phases. In contrast, BCrGO samples (Figure 6g–i) display comparatively smoother surfaces with clear layered features, indicating partial structural restoration after reduction.

**Figure 6:**
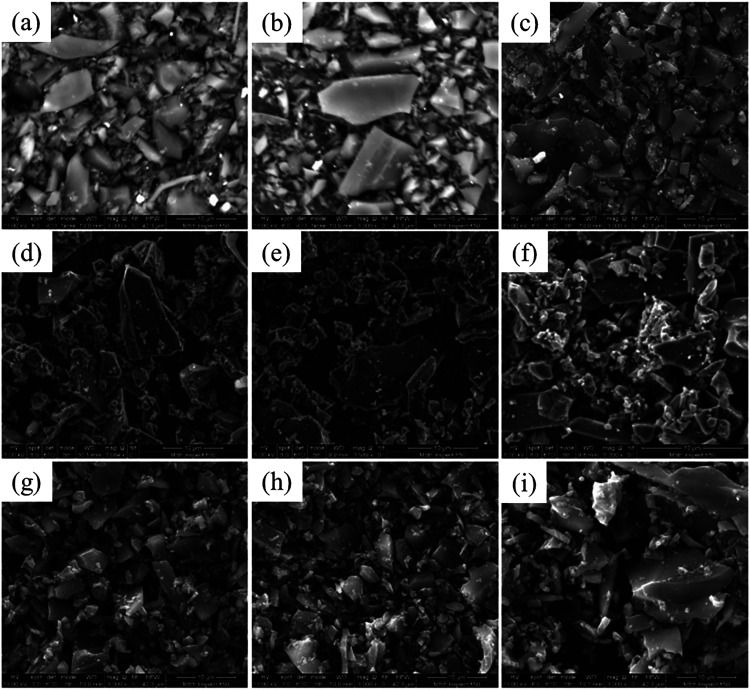
SEM micrographs for: (a) BC450, (b) BC600, (c) BC900, (d) BCGO450, (e) BCGO600, (f) BCGO900, (g) BCrGO450, (h) BCrGO600, and (i) BCrGO900. Scale -10 μm.

### 5.2 Cement Paste Evaluation

#### 5.2.1 Isothermal Calorimetric Analysis and Degree of Hydration

A comparative calorimetric study was conducted on cement paste samples to evaluate the influence of BCGO and BCrGO on cement hydration over 72 h, as shown in Figure 7. Figure 7a,b presents the heat flow and degree of hydration (α), respectively. As shown in Figure 7a, all pastes exhibited the five characteristic hydration stages of Portland cement: initial reaction, induction, acceleration, deceleration, and decline.^61^ It is evident that incorporating BCGO and BCrGO at different pyrolysis temperatures did not introduce or eliminate hydration peaks; instead, it modified their intensities. Within the first 24 h, the heat-flow curves exhibited an initial dissolution peak at very early ages, followed by the main acceleration peak due to C_3_S hydration at approximately 7 to 8 h and a subsequent aluminate-related shoulder between 8 and 10 h arising from sulfate depletion.^62^ The degree of hydration (α) curve in Figure 7b represents the extent of cement reaction with water, leading to the formation of hydration products such as CSH and calcium hydroxide (CH), which govern concrete strength development.^63^ This was calculated from the cumulative heat release obtained from isothermal calorimetry and expressed as a percentage of the total reaction.

**Figure 7:**
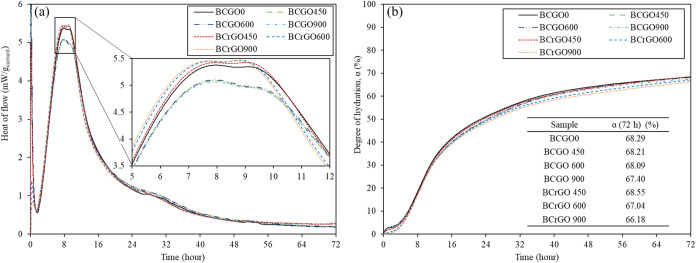
Heat of hydration of cement paste: (a) Heat of flow, and (b) degree of hydration.

From the inset of Figure 7a, the control paste (BCGO0) exhibited a higher main silicate hydration peak at 7.95 h with a heat flow of 5.38 mW g^–1^, corresponding to an α of 18.22% and a cumulative heat of 86.47 J g^–1^ (Figure 7b). All BCGO pastes showed a similar peak sequence, with the first peak higher than the second but with reduced intensities (5.06–5.10 mW g^–1^ and an α value of approximately 17.24 to 17.29%) relative to the control. In contrast, BCrGO pastes exhibited variations in early peak heat flows (5.43–5.45 mW g^–1^), along with differences in cumulative heat (75–84 J g^–1^) and degree of hydration (15.72–17.64%). The second subpeak, occurring between approximately 8.5 and 9.1 h, is associated with aluminate phase reactions following sulfate depletion and the conversion of C_3_A to ettringite (AFt).^64^ Over 72 h, BCGO systems showed a degree of hydration of approximately α_72h_ of 68%, while BCrGO systems showed approximately an α_72 h_ of 67%, indicating only minor differences between the systems. Considering the limited variation observed, these results suggest that incorporating biochar-derived graphene materials does not significantly alter the fundamental hydration mechanism of cement but rather affects the hydration kinetics within a narrow range. Differences between BCGO and BCrGO may be associated with variations in surface characteristics. GO contains a higher amount of oxygenated functional groups,^65–67^ which can interact with water and the cementitious system, whereas rGO has a more reduced structure with fewer such groups.^68,69^ As a result, GO may interact more with the mixing water, while rGO interacts to a comparatively lesser extent. These differences in surface functionality may contribute to the observed variations in peak intensity and hydration behavior. However, given the inherent heterogeneity of both cement and biochar-derived materials, as well as the small magnitude of these differences, the results should be interpreted as indicative trends rather than definitive changes in hydration behavior.

#### 5.2.2 X-ray Fluorescence (XRF) Analysis

XRF analysis was conducted on cement paste to examine the variation in oxide composition resulting from the incorporation of BCGO and BCRGO at different curing ages (7 and 28 days of curing). The major oxides detected were CaO, SiO_2_, Al_2_O_3_, and SO_3_, with minor quantities of K_2_O, TiO_2_, Fe_2_O_3_, P_2_O_5_, and MgO. Compared with the control cement paste, all modified pastes exhibited an apparent increase in the relative CaO content and a substantial reduction in the relative SiO_2_ and SO_3_ contents with an increasing curing age. The CaO content increased from about 63% in the control (**BCGO0**) to more than 80% in most 28-day mixes, particularly in BCrGO600 (83.38%) and BCrGO900 (82.36%), while SiO_2_ decreased from its initial level of 15–19% at 7 days to below 8% at 28 days across all mixes. SO_3_ values declined from approximately 2–3% to nearly 0% by 28 days, indicating near-complete consumption of sulfate-bearing phases (gypsum and ettringite stabilization). It is important to note that these XRF data represent normalized bulk oxide compositions measured on the specimen surface, rather than the absolute stoichiometry of individual hydration products. During hydration, CaO, SiO_2_, and SO_3_ all participate in the formation of CH, CSH, and ettringite; however, the depletion of Si- and S-bearing clinker phases, together with the formation of Ca-rich hydration products, results in a relative increase in CaO in the normalized XRF spectrum at 28 days. Therefore, the observed increase in CaO does not indicate a lack of calcium consumption but rather reflects changes in the relative oxide proportions within the analyzed surface region as hydration progresses.

The elemental ratios derived from this composition, Ca/Si, SO_3_/Al_2_O_3_, and Ca/(Si+Al), provide further insight into the evolution of the bulk chemistry and are summarized in Table 4. As these ratios are calculated from normalized oxide contents, they should be interpreted only as indicators of relative compositional changes rather than direct measures of phase development.^70^ From 7 to 28 days, Ca/Si and Ca/(Si+Al) ratios consistently increased for all BCGO- and BCrGO-incorporated samples. Early age values ranged between 3.6 and 5.4 for BCGO samples, while BCrGO systems reached higher ratios (up to 9.06 for BCrGO600), suggesting faster clinker dissolution and accelerated hydration. At 28 days, the ratios rose sharply, particularly for BCrGO600 and BCrGO900 (22.99 and 21.47, respectively), indicating extensive calcium enrichment. The SO_3_/Al_2_O_3_ ratios approached zero by 28 days, indicating complete sulfate consumption. The BCrGO mixes showed the highest overall calcium enrichment and the lowest residual of SO_3_, confirming their reactivity and link to the higher cumulative heat and degree of hydration trends in Figure 7. In contrast, BCGO at 900 °C exhibited lower ratios due to fewer oxygenated functional groups, consistent with its lower hydration heat in Figure 7. These overall XRF results demonstrate that BCrGO provides a more favorable chemical environment for efficient hydration and paste densification than BCGO.

**Table 4:** XRF Analysis on Cement Paste

**sample ID**	**curing ages (days)**	**Ca/Si**	**SO**_**3**_ **/Al**_**2**_ **O**_**3**_	**Ca/(Si+Al)**
BCGO0	7	3.57	0.71	2.96
	28	10.48	0.00	6.74
BCGO450	7	4.05	0.68	3.29
	28	11.57	0.04	7.56
BCGO600	7	4.78	0.42	3.61
	28	10.95	0.00	6.62
BCGO900	7	4.64	0.46	3.52
	28	7.36	0.00	4.97
BCrGO450	7	5.38	0.46	3.91
	28	14.37	0.00	7.29
BCrGO600	7	9.06	0.32	5.75
	28	22.99	0.00	10.14
BCrGO900	7	6.44	0.51	4.70
	28	21.47	0.00	9.43

#### 5.2.3 X-ray Diffraction (XRD) Analysis

Figure 8 shows the XRD patterns of the cement pastes by indicating the major hydration phases of Portland cement, including CH, CSH, ettringite (AFt), calcite (CaCO_3_), and unhydrated belite (C_2_S). Compared with the control paste (BCGO0), no new diffraction peaks are observed after introducing WB materials into the cement paste; instead, only changes in the peak intensities are evident. This suggests that the addition of BCGO and BCrGO does not significantly alter the bulk-phase composition of the hydrated paste. Instead, their primary influence likely occurs at the nanoscale by modifying hydration kinetics and the microstructure of the CSH phase rather than producing new crystalline products. This interpretation aligns with the heat of hydration results, which show only modest differences in early age hydration behavior among the mixes, and with the XRF results, where changes in CaO, SiO_2_, and SO_3_ are described as relative shifts in normalized oxide compositions associated with ongoing hydration rather than as evidence of new crystalline phases.

**Figure 8:**
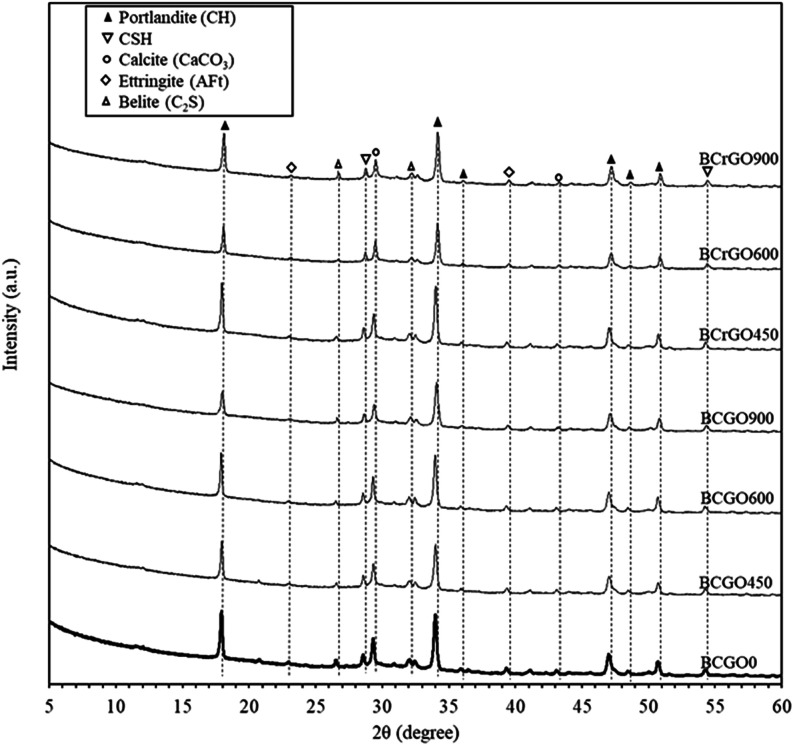
XRD analysis on cement paste.

#### 5.2.4 Scanning Electron Microscopy (SEM) Coupled with Energy-Dispersive X-ray Spectroscopy (EDS) Analysis

Figure 9 presents SEM micrographs and EDS elemental maps of the 28-day hydrated cement pastes. Images were acquired at magnifications corresponding to 5 and 1 μm scales, highlighting the overall microstructural morphology and nanoscale hydration features, respectively. The corresponding nanoscale (1 μm) EDS elemental mappings and spectra confirm the spatial distribution of calcium (Ca K), silicon (Si K), aluminum (Al K), and carbon (C K) across the cement paste, where “K” denotes the characteristic K-shell (Kα) X-ray emission used for elemental identification.

**Figure 9:**
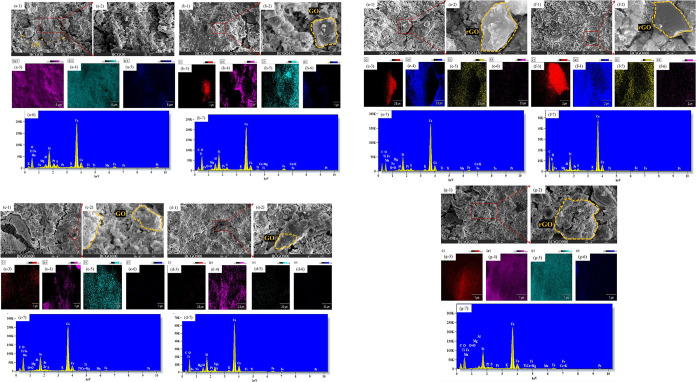
SEM micrographs and EDS elemental mappings of hydrated cement pastes: (a) BCGO0, (b) BCGO450, (c) BCGO600, (d) BCGO900, (e) BCrGO450, (f) BCrGO600, and (g) BCrGO900. Subpanels show low- and high-magnification SEM images, corresponding C, Ca, Si, and Al elemental maps, and EDS spectra acquired from the same analyzed regions.

Because of the limited magnification and resolution of the SEM images, the comparisons between samples are primarily based on qualitative visual observations. The control paste (BCGO0) [Figure 9(a-1)–(a-6)] exhibits a relatively rough and porous surface structure with visible CH plates as indicated by the yellow arrow in Figure 9(a-1). The elemental area mapping in Figure 9(a-6) shows a homogeneous Ca-rich surface. Upon BCGO incorporation, the morphology becomes progressively denser, and the surface appears smoother. The BCGO450 paste [Figure 9(b-1)–(b-7)] shows the formation of CSH in a cloud-like amorphous morphology over the BCGO sheets (highlighted in yellow dashed outlines). The elemental area mapping reveals Ca and Si signals with overlapping C-rich regions, confirming chemical affinity between oxygenated BCGO sites and cement hydrates. This enhanced CSH nucleation is consistent with the calorimetry results in Figure 7a, which shows that BCGO450 exhibited higher hydration kinetics. In the BCGO600 and BCGO900 samples [Figure 9(c-1)–(d-7)], the surfaces appear smoother with a slightly reduced fibrous morphology, which can be attributed to the partial reduction of oxygen-containing functional groups. For the BCrGO-incorporated pastes [Figure 9(e-1)–(g-7)], the microstructure shows a relatively rough surface similar to the control mix. This behavior is associated with the reduced interaction between the cement paste and WB materials. On the basis of the material characterization, rGO contains fewer oxygen functional groups compared to GO, which limits the nucleation and growth of hydration products on its surface. As a result, hydration products tend to form independently and fill surrounding voids rather than forming a bonded structure on the rGO surface.

Another observation is noted for the higher pyrolysis temperature samples (BCGO900 and BCrGO900), where the WB structures appear more fragmented and show increased attachment of hydration products on and around their surfaces compared with lower-temperature materials. Among these, BCrGO900 exhibits a more pronounced clustering of hydration products around the WB material.

#### 5.2.5 Fourier-Transform Infrared Spectroscopy (FT-IR) Analysis

Figure 10 displays the FT-IR spectra of hydrated cement pastes, highlighting vibrational features of key hydration products. For qualitative comparison of hydration-related phase evolution, only a limited number of representative samples were selected for FT-IR analysis. The bands at 3640 and 3400 cm^–1^ (O–H stretching) and 1650 cm^–1^ (H–O–H bending) represent chemically bound and adsorbed water, primarily in portlandite (CH).^71^ The prominent region between 1108 and 950 cm^–1^ corresponds to Si–O–Si and Si–O–Ca stretching in the CSH phase, while peaks at 1420, 870, and 700 cm^–1^ indicate minor carbonation.^71^ In this study, spectral interpretation is restricted to wavenumbers above approximately 400 cm^–1^ because features at lower wavenumbers lie at the edge of the instrument’s reliable range and are attributed to edge artifacts rather than distinct vibrational modes of cement hydration products. Increased peak intensity and sharpness, especially in the silicate region, signify higher concentrations or greater structural organization of these phases, which directly correlate with mechanical strength development.

**Figure 10:**
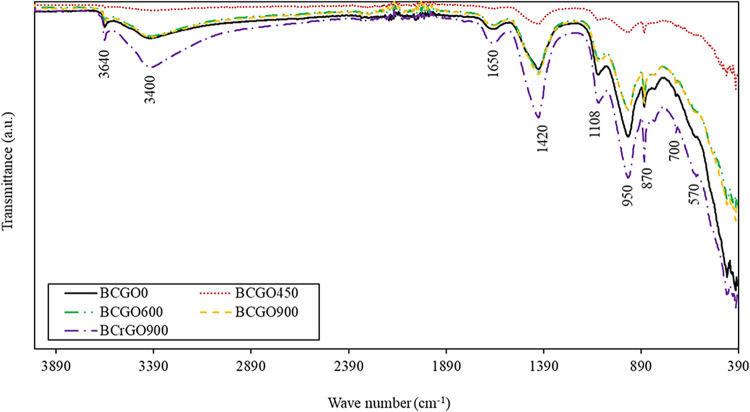
FT-IR analysis on cement paste.

The control paste (BCGO0) exhibits baseline hydration features characterized by moderate absorption across the O–H- and C–S–H-related regions.^72^ Upon incorporation of WB materials, the spectral response varies with pyrolysis temperature. BCGO450 exhibits an evident shift in absorption features, which is due to oxygen-containing functional groups of BCGO retained at lower temperatures, facilitating altered chemical interactions and phase redistribution within the cement paste. Conversely, BCGO600 and BCGO900 show overlapping spectra with moderate intensities, indicating that the reduction of surface functional groups at higher temperatures leads to a more stabilized, but less chemically active, hydration response. The most intense and sharpest absorption features occur in the BCrGO900 paste. This increased sharpness suggests a highly ordered hydration environment, likely driven by the structural ordering of BCrGO, which acts as a physical nucleation template for the spatial arrangement of hydration products. The two distinct spectral behaviors of BCGO450 and BCrGO900 are consistent with higher heat evolution and a greater degree of hydration, as observed in the calorimetric results in Figure 7.

### 5.3 Fresh and Hardened Concrete Properties

#### 5.3.1 Workability of Concrete (Slump Test)

Figure 11 presents the slump behavior of concrete incorporating BCGO [Figure 11a] and BCrGO [Figure 11b] additives at 0.05 and 0.5% dosages, including a control mix, which represents the workability of freshly mixed concrete.^73^ The control mix showed a slump of 155 mm, whereas BCGO-incorporated concrete ranged from 91 to 163 mm and BCrGO mixes from 89 to 147 mm, indicating a general reduction with increasing WB additive content. At 0.05%, the average slump reduction was approximately 25–35% for BCGO and 20–30% for BCrGO, whereas at 0.5%, the reduction increased to 40–45% and 35–40%, respectively.

**Figure 11:**
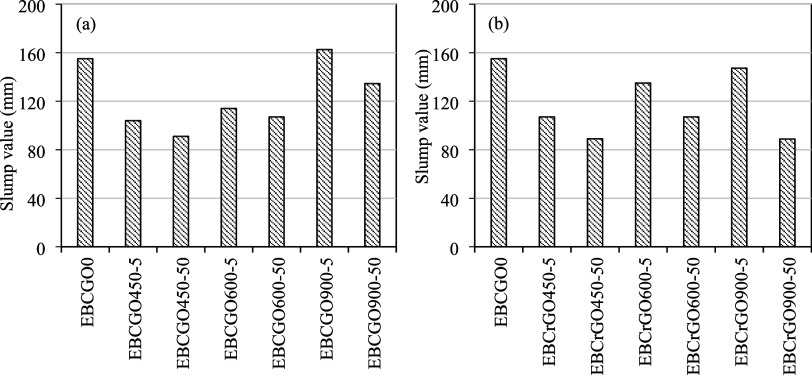
Concrete consistency test: (a) GO mixed concrete; (b) rGO mixed concrete.

Both WB materials showed a clear trend of increasing slump with higher pyrolysis temperature. For instance, in the BCGO series, the 900 °C concrete mix exhibited nearly 70% higher slump than the 450 °C mix at a 0.05% dosage, whereas in the BCrGO series, the increase was approximately 38%. This pattern, based on pyrolysis temperature, indicates that elevated synthesis temperatures improve workability by lowering surface polarity and reducing water adsorption capacity.^74^ Across the WB materials, increasing the dosage of both BCGO and BCrGO exhibits a similar overall pattern of slump reduction, which is in line with previous studies on graphene- ^12,16^ and coal-derived^9^ GO and rGO. In those studies, increased surface area and the entrapment of water within the nanocarbon network of graphene derivatives were reported to increase the effective water demand of the mix, thereby reducing the measured slump.

#### 5.3.2 Density Analysis

The 28-day densities of the BCGO- and BCrGO-mixed concrete are shown in Figure 12a,b, respectively. The control mix exhibited an average density of 2.26 ± 0.02 g/cm^3^, whereas BCGO- and BCrGO-incorporated concrete showed slightly higher densities of approximately 2.35–2.37 and 2.32–2.39 g/cm^3^, respectively. The marginal rise in density (3–5%) suggests that WB materials at lower dosages can improve particle packing and reduce entrapped air by filling fine voids between cement hydrates.^75^ This behavior is supported by SEM observations (Figure 9), which shows that WB materials, being significantly smaller than cement particles, not only fill voids but also promote the formation of a denser CSH network around them. The resulting hydration matrix is therefore expected to exhibit fewer capillary voids in WB-incorporated paste, indicating improved packing of hydration products. When comparing BCGO with BCrGO at equivalent pyrolysis temperatures, BCrGO-incorporated concrete exhibited slightly higher densities. This increase in density aligns with the lower C/O ratio observed in XPS analyses in Figure 5, reflecting a more graphitized and less oxygen-functionalized carbon framework, reducing water adsorption, swelling, and nanosheet agglomeration during hydration, thereby enabling closer packing of carbon sheets and promoting more uniform CSH growth at the nanoscale.^16^

**Figure 12:**
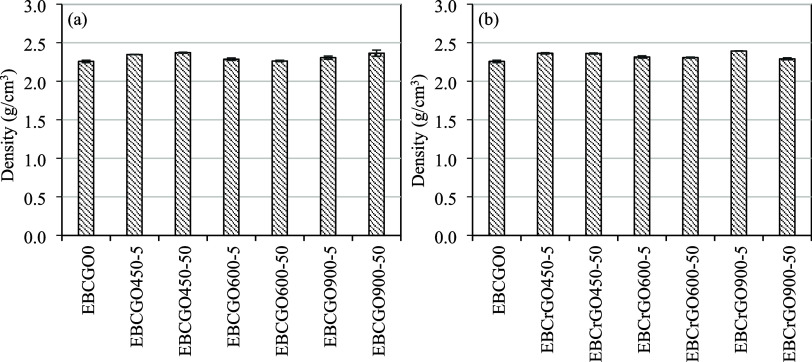
Density test of concrete: (a) BCGO; (b) BCrGO.

#### 5.3.3 pH Test

The pH of concrete using OPC typically lies between 12.5 and 13.0, governed by the dissolution of alkali hydroxides (NaOH and KOH) and calcium hydroxide (Ca­(OH)_2_) formed during hydration.^76^ In this study, the 28-day control mix exhibited a pH of 12.6, consistent with the abovementioned range for regular concrete, while the BCGO- and BCrGO-mixed concrete showed slightly higher values of 13.4 to 13.5 across all dosages and pyrolysis temperatures, as shown in Table 5. This moderate increase in alkalinity can be attributed to the accelerated hydration kinetics and enhanced CH formation induced by BCGO and BCrGO.^77^ The oxygenated functional groups (−OH, −COOH, −C=O) present on BCGO and partially retained on BCrGO surfaces interact with Ca^2+^ ions in the pore solution, stimulating nucleation of CSH and facilitating clinker dissolution.^78^ This interaction is supported by XRD (Figure 3) and XPS (Figure 5) results showing a higher density of oxygen-containing functional groups and surface oxygen in BCGO compared to BCrGO, which promotes Ca^2+^ adsorption and CSH nucleation, increasing hydroxyl ion concentration, and thereby raising the pore solution pH, indicative of a more mature hydration environment.^79^

**Table 5:** pH of Concrete Samples

	**pH**	
**temperature (**°**C) – WB (%)**	**BCGO**	**BCrGO**
control mix	12.6	
450–0.05	13.4	13.5
450–0.50	13.5	13.4
600–0.05	13.4	13.5
600–0.50	13.4	13.4
900–0.05	13.5	13.4
900–0.50	13.5	13.5

A slightly higher pH environment is generally beneficial to concrete performance. It sustains the passive oxide film that protects steel reinforcement and stabilizes hydration products such as CSH.^80^ Within moderate limits, a higher pH also reflects more complete hydration and greater availability of CH, both of which can be correlated with increased compressive and elastic properties in concrete.^78^

#### 5.3.4 XRD Analysis

Figure 13 illustrates the XRD patterns of BCGO- and BCrGO-incorporated 28-day concretes. The diffraction peaks correspond to crystalline phases of CH, CSH, C_2_S, and C_3_S.^81^ All mixes exhibit primary hydration phases similar to those of the control mix. In particular, compared with the control mix (EBCGO0), the intensities of the CH peaks (18 and 34°) show slight variations in BCGO- and BCrGO-incorporated concretes, with generally higher intensities observed in mixes with higher dosage. The increased sharpness of the CSH-related peaks between 27 and 34° suggests improved hydration gel densification in BCGO- and BCrGO-incorporated concretes.^82^ A temperature-dependent trend is also evident in both the BCGO and BCrGO systems; the 450 °C composites exhibit more pronounced CSH and C_3_S peaks than those prepared at 900 °C. At higher pyrolysis temperatures, partial reduction and loss of functional groups reduce reactivity, resulting in diminished CH consumption. These XRD observations are consistent with the XRF-derived Ca/Si enrichment in Table 4 and with the enhanced hydration kinetics observed by calorimetric analysis in Figure 7, collectively confirming the role of BCGO and BCrGO in promoting hydration and paste densification.

**Figure 13:**
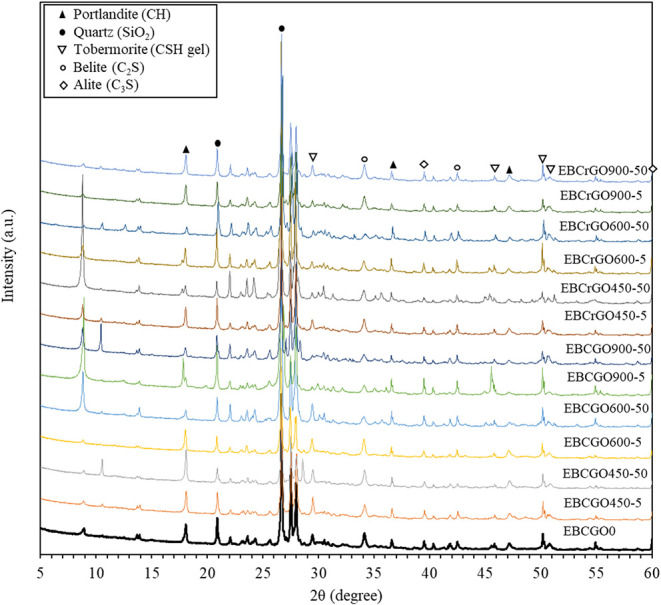
XRD analysis of concrete samples reveals both anhydrous and hydrated phases.

#### 5.3.5 Uniaxial Compressive Strength (UCS) Test

Figure 14 illustrates the compressive strength development of BCGO- (Figure 14a) and BCrGO-incorporated (Figure 14b) concretes from 7 to 56 days. In the BCGO-incorporated concrete batches (Figure 14a), low-temperature BCGO shows a higher compressive strength in all curing days. This EBCGO450–50 achieved approximately 9.8% higher UCS at 28 days and 8.3% at 56 days compared to the control mix. This enhancement is attributed to the abundance of oxygenated functional groups identified by FT-IR (Figure 10) and XPS (Figure 5), which promote clinker dissolution and refinement of hydration products.^83^ EBCGO600 resulted in strength reductions of about 4–6% at 56 days, while a moderate recovery was observed from EBCGO900, where the 0.5% dosage produced strength gains of 2.1% at 28 days and 6.6% at 56 days, corresponding to slower early but sustained hydration at later ages.

**Figure 14:**
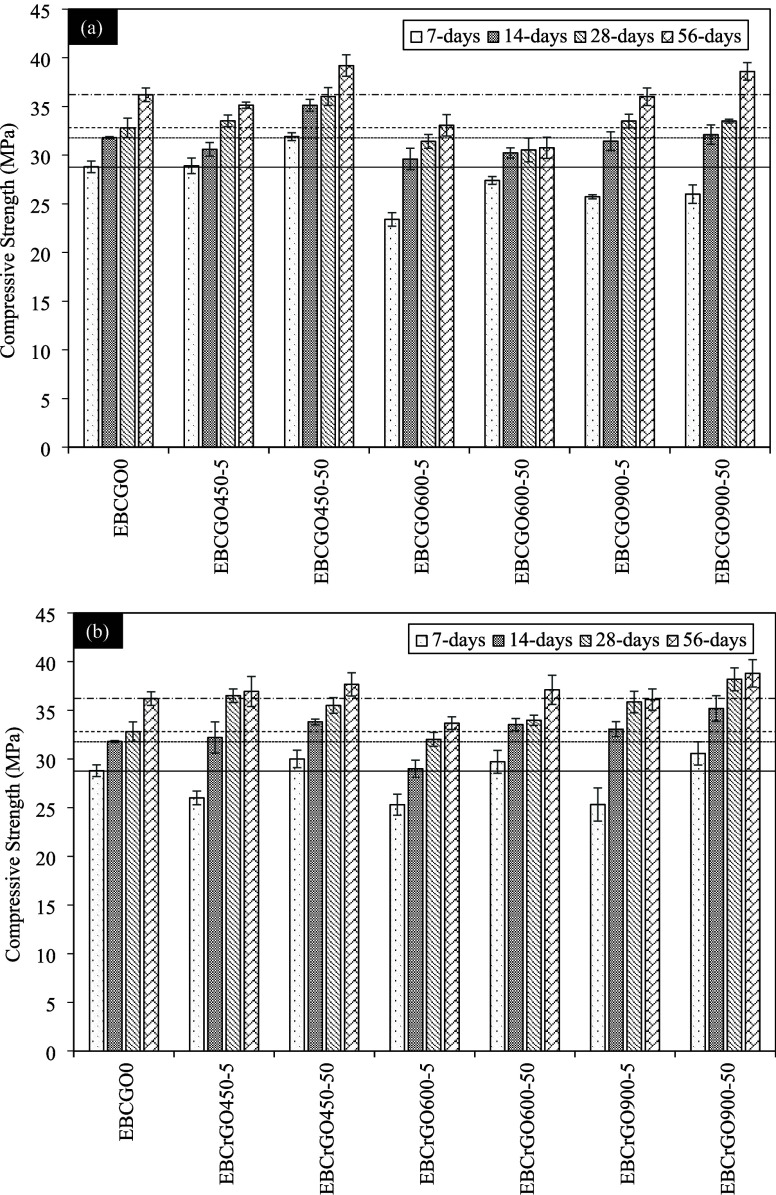
Compressive strength test: (a) BCGO and (b) BCrGO.

In contrast, BCrGO concretes exhibited the opposite temperature-dependent trend. EBCrGO900 yielded the highest gain in compressive strength, with the 0.5% dosage achieving 16.4% at 28 days and 7.2% at 56 days relative to the control mix. This was followed by the EBCrGO450 mixes, which showed moderate strength gains at both dosages compared with the EBCrGO600 concrete at 28 and 56 days, although the magnitude of increment decreased with the curing age. These results indicate that in BCrGO-incorporated concrete, residual oxygenated functional groups primarily contribute to early age hydration enhancement, while their influence diminishes at later ages.

When compared directly, BCrGO typically outperformed BCGO-incorporated concrete under equivalent temperature and dosage conditions. This behavior is attributed to controlled surface activity and enhanced structural stability of BCrGO, as confirmed by XRD analysis (Figure 3), which collectively promote enhanced hydration, as evidenced by heat of hydration and degree of hydration results (Figure 7). These findings are consistent with previous studies reporting that controlled reduction of BCGO enhances hydrophilicity and concrete densification, leading to improved mechanical performance of cementitious composites.^64,84^

#### 5.3.6 Flexure and Indirect Split Tensile Strength Tests

The 28-day flexural strength results for BCGO- and BCrGO-incorporated concretes are shown in Figure 15a. The control mix (EBCGO0) achieved 4.5 ± 0.30 MPa, while BCGO-mixed concrete exhibited a modest reduction or comparable strength depending on pyrolysis temperature and dosage. The EBCGO900 series showed slightly higher performance than other EBCGO samples; EBCGO900–5 was 7% lower than the control, and EBCGO900–50 nearly matched the control. The BCrGO-incorporated concrete exhibited generally higher flexural strengths than their BCGO counterparts across all temperatures and dosages. The EBCrGO450–5 achieved the highest increase of approximately 4% relative to the control, indicating improved crack bridging and load transfer due to enhanced sheet integrity and graphitic ordering, as described earlier in the characterization. At elevated pyrolysis temperatures (600 to 900 °C), BCrGO-incorporated concrete maintained a stable strength (4.3 to 4.5 MPa) with minimal variation across dosages, comparable to the control mix.

**Figure 15:**
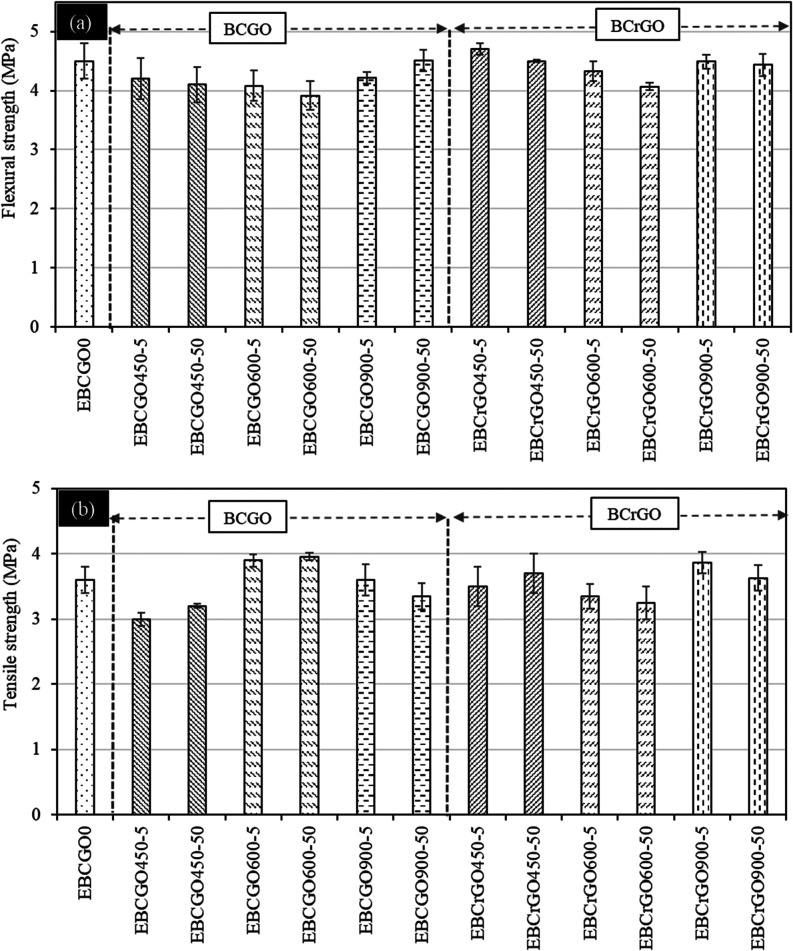
(a) Flexural strength and (b) indirect split tensile strength tests of concrete samples.

Figure 15b illustrates the 28-day tensile strength results of WB-incorporated concrete. Among BCGO-incorporated concrete, EBCGO600 exhibited the best performance, with EBCGO600–5 and EBCGO600–50 achieving 8–11% gains over the control mix. In contrast, BCrGO-incorporated concretes did not exhibit a distinct peak improvement at any specific temperature or dosage. Instead, they showed more uniform tensile performance across all temperatures and dosages, with variations generally confined within ±10% of the control mix; among them, BCrGO450 and BCrGO900 exhibited slightly higher tensile improvements compared with BCrGO600. When directly compared at the same pyrolysis temperature, an opposite trend was observed between the BCGO and BCrGO systems. At 600 °C, BCGO-incorporated concrete outperformed BCrGO600 in tensile strength, whereas at 450 and 900 °C, BCrGO-incorporated concrete showed relatively higher tensile performance than the corresponding BCGO-incorporated concrete. This behavior indicates that the higher oxygen content of BCGO plays a critical role in enhancing tensile load transfer at intermediate pyrolysis temperatures by improving interfacial bonding and regulating hydration.^68^ Conversely, the more graphitic and less functionalized structure of BCrGO contributes to stable but less dosage-sensitive tensile behavior.

According to previous studies, the improvement in flexure and tensile strength of concrete incorporating graphite-derived GO or rGO generally occurs at low dosages (≤0.1% BWOC).^64,85^ This study also observed the same trend, in comparison between WB materials, at low dosages of BCGO, yielding the most significant gains in tensile and flexural strength due to its ability to regulate hydration crystal growth, promote the formation of flower-like CSH structures, and improve toughness through its template effect.^86^ However, at higher WB dosages, the observed reduction in strength is likely due to poor dispersion and the formation of weak zones.^87^

#### 5.3.7 Elastic Behavior Test (Modulus of Elasticity and Poisson’s Ratio)

The modulus of elasticity (MOE) and Poisson’s ratio of BCGO- and BCrGO-incorporated concretes are summarized in Table 6, representing the elastic behavior of WB-based concrete. Relative to the control mix, all WB-incorporated concrete exhibited increases in MOE, with enhancements generally ranging from approximately 35 to 55%. At lower dosages (0.05%), MOE gains were typically in the range of about 40–50%. In comparison, higher dosages (0.50%) maintained comparable improvements, indicating that stiffness enhancement was not strongly dosage-sensitive within the investigated range. Across pyrolysis temperatures, both BCGO and BCrGO showed consistent MOE refinement. In contrast, Poisson’s ratio remained relatively stable, varying only between 0.15 and 0.16, suggesting that the addition of WB materials significantly enhanced elastic stiffness without altering the overall deformation characteristics of the concrete.

**Table 6:** Elastic Behavior of Concrete Samples

	**modulus of elasticity (GPa)**		**Poisson’s ratio**	
**temperature (°C) – BCGO/BCrGO (%)**	**BCGO**	**BCrGO**	**BCGO**	**BCrGO**
control mix	14.85 ± 0.62		0.16 ± 0.01	
450–0.05	22.64 ± 0.16	22.62 ± 1.05	0.16 ± 0.00	0.15 ± 0.00
450–0.50	22.30 ± 0.65	21.97 ± 0.96	0.16 ± 0.01	0.15 ± 0.00
600–0.05	20.08 ± 0.57	23.68 ± 1.66	0.16 ± 0.00	0.16 ± 0.00
600–0.50	20.73 ± 2.56	21.19 ± 0.63	0.16 ± 0.00	0.16 ± 0.00
900–0.05	21.16 ± 0.86	20.53 ± 0.76	0.16 ± 0.00	0.16 ± 0.00
900–0.50	20.66 ± 1.42	22.26 ± 3.33	0.18 ± 0.01	0.16 ± 0.00

This MOE refinement is attributed to the nanofiller and nucleation effects of BCGO and BCrGO, which enhance cement hydration and densify the microstructure through the formation of compact CSH gels. The oxygenated functional groups of BCGO improve interfacial bonding and stress transfer, while the partially reduced structure of BCrGO enhances load-bearing continuity via increased sp^2^ carbon domains. However, in concrete, the modulus of elasticity reflects the combined response of the aggregate–paste composite system. Since the coarse aggregates were identical in all mixtures, the observed increase in MOE is primarily associated with improvements in the cement paste and, in particular, the interfacial transition zone (ITZ), which enhances stress-transfer efficiency between paste and aggregates rather than altering the intrinsic stiffness of the aggregates themselves.^85,87–89^ Earlier studies have shown that the elastic modulus can increase by approximately 20% (e.g., from ≈19.9 to ≈23.9 GPa), while the corresponding strength gains remain comparable.^14,90^ This difference arises because the modulus of elasticity is governed by the material response at low strain levels, where stiffness and microstructural continuity dominate, whereas compressive and flexural strengths are controlled by crack initiation and propagation at higher strain levels. As a result, improvements in stiffness do not necessarily translate into proportional increases in strength.

## 6 Conclusions

This study demonstrates the feasibility of synthesizing graphene derivatives from pine sawmill waste biomass, specifically biochar-based graphene oxide (BCGO) and reduced graphene oxide (BCrGO), using an alternative nitric-acid–based method, and evaluates their effectiveness in cementitious composites. The principal conclusions are as follows:

(1) Biochar-based graphene oxide (BCGO) and reduced graphene oxide (BCrGO) were successfully synthesized from waste pinewood biomass at pyrolysis temperatures of 450, 600, and 900 °C. XRD, Raman spectroscopy, XPS, and SEM analyses confirmed the formation of partially layered carbon nanostructures with temperature-dependent oxygen functionality and graphitic order. The precursor pyrolysis temperature provided effective control over surface chemistry and structural ordering, enabling graphene derivatives suitable for cementitious applications.
(2) At the cement paste scale, WB-incorporated systems exhibited comparable early age hydration behavior. Isothermal calorimetry showed that BCGO- and BCrGO-incorporated pastes had slightly different heat-flow peak characteristics while exhibiting very similar overall degrees of hydration, indicating only modest differences in hydration kinetics associated with different WB materials. XRD and XRF analyses confirmed that WB incorporation did not introduce new hydration phases, but instead resulted in relative changes in peak intensities and oxide compositions of existing phases, consistent with ongoing hydration reactions, including the formation of hydration products and the progressive consumption of silicate and sulfate phases. FT-IR and SEM observations further demonstrated strengthened Si–O–Si and Si–O–Ca bonding environments, along with nucleation-driven microstructural refinement and densification in the hydrated paste.
(3) At the concrete scale, the incorporation of BCGO and BCrGO resulted in consistent improvements in mechanical and elastic properties relative to the control mix. Compressive strength increased by up to 17%, while flexural and tensile strengths improved by up to 7 and 10%, respectively. The modulus of elasticity increased by as much as 55%, indicating substantial stiffening of the composite. Despite an approximately 5% increase in density, Poisson’s ratio remained essentially unchanged, suggesting that enhanced stiffness was achieved without compromising ductility. Measured pH values (∼13.5) confirmed the maintenance of a stable alkaline environment favorable for long-term durability.
(4) BCGO450 and BCrGO900 at 0.5% BWOC consistently exhibited comparatively higher performance across the measured properties. This behavior illustrates the importance of the precursor biochar material properties and their influence on the resulting GO and rGO products, as drastic variability in performance is observed among the WB materials. This is evidenced by the fact that the two most dissimilar materials – BCGO450 being the least intensively processed sample while BCrGO900 is the most – resulted in the highest performing admixtures, suggesting that multiple properties of the additives influence concrete in different ways. In BCGO450, the performance increase is likely driven by high oxygen content facilitating hydration, while a more defined structure likely fills this role for BCrGO900, which has little to no oxygen content. This behavior suggests a favorable balance between surface oxygen functionality, which promotes chemical interaction with hydration products, and graphitic structural integrity, which supports efficient mechanical load transfer. Overall, the results of this work demonstrate the successful implementation of an alternative nitric acid method for the synthesis of GO and rGO, thereby enhancing the engineering properties of concrete.
(5) The effectiveness of the materials studied introduces a low-cost alternative to conventional GO and rGO materials for use in concrete admixtures. With initial estimates suggesting a price of less than $30/kg for GO and less than $50/kg for rGO compared with well over $1000/kg for commercial options, these materials open the door to potential large-scale adoption of graphene derivatives in the concrete industry.

## Data Availability

The original contributions presented in this study are included in the article.

## References

[1] Stefaniuk D., Hajduczek M., Weaver J. C., Ulm F. J., Masic A. (2023). Cementing
CO2 into C-S-H: A Step toward Concrete Carbon Neutrality. PNAS Nexus.

[2] Miao R. K., Wang N., Hung S.-F., Huang W.-Y., Zhang J., Zhao Y., Ou P., Wang S., Edwards J. P., Tian C., Han J., Xu Y., Fan M., Huang J. E., Xiao Y. C., Ip A. H., Liang H., Sargent E. H., Sinton D. (2023). Electrified Cement Production via
Anion-Mediated Electrochemical Calcium Extraction. ACS Energy Lett..

[3] Islam Md. J., Dipta I. A., Rahat M. (2018). Investigation
of Recycled Poly-Ethylene
Terephthalate (PET) as Partial Replacement of Coarse Aggregate in
Concrete. J. Civil Eng..

[4] Sarker M. H. U., Tamjeed A., Dipta I. A., Rahman K. T., Bhuya E. A., Islam M. S. (2025). Characterization
and Performance Evaluation of Thermally
Treated Black Tea Waste as Partial Fine Aggregate Replacement in Concrete. Next Sustainability.

[5] Adesina A. (2020). Recent Advances
in the Concrete Industry to Reduce Its Carbon Dioxide Emissions. Environ. Challenges.

[6] Dipta I. A., Ng K., Lau C. K., Yu H. (2025). Coal-Derived
Char for Improving Mechanical
Performance and Microstructural Characteristics of Concrete. Journal of Sustainable Cement-Based Materials.

[7] Chadwick, J. C. ; Dipta, I. A. ; Johnson, P. ; Ng, K. W. Concrete and Cementitious Compositions Containing Graphene Oxide. AU2024210107A1, July 17, 2025.

[8] Ng, K. W. ; Dipta, I. A. Coal-Derived Carbon-Based Concrete and Methods of Making the Same. WO2025151868A1, July 17, 2025.

[9] Dipta I., Ng K., Chadwick J., Lau C. K., Yu H., Johnson P. A. (2025). Effect
of Coal-Derived Graphene Oxide on the Mechanical and Microstructural
Characteristics of Concrete. Materials.

[10] Devi S. C., Khan R. A. (2020). Effect of Graphene
Oxide on Mechanical and Durability
Performance of Concrete. J. Build. Eng..

[11] Chuah S., Pan Z., Sanjayan J. G., Wang C. M., Duan W. H. (2014). Nano Reinforced
Cement and Concrete Composites and New Perspective from Graphene Oxide. Constr. Build. Mater..

[12] Akarsh P. K., Shrinidhi D., Marathe S., Bhat A. K. (2022). Graphene Oxide as
Nano-Material in Developing Sustainable Concrete – A Brief
Review. Mater. Today: Proc..

[13] Gao Y., Zou F., Wang S., Sui H., Yu J., Xu B., Chen W., Liu Y. (2025). Redefining
the Cement Substitution
Potential of Recycled Concrete Powder Using Graphene Oxide Coating. Cem. Concr. Compos..

[14] Dipta I. A., Shukla V., Ferreira J. M., Lau C. K., Ng K., Johnson P. A. (2025). Comparative
Evaluation of Coal-Derived and Commercial
Graphene Oxides in Concrete Strength Performance. ACS Appl. Eng. Mater..

[15] Jing G., Wu J., Lei T., Wang S., Strokova V., Nelyubova V., Wang M., Ye Z. (2020). From Graphene Oxide to Reduced Graphene
Oxide: Enhanced Hydration and Compressive Strength of Cement Composites. Constr. Build. Mater..

[16] Valizadeh
Kiamahalleh M., Gholampour A., Tang Y., Ngo T. D. (2024). Incorporation
of Reduced Graphene Oxide in Waste-Based Concrete Including Lead Smelter
Slag and Recycled Coarse Aggregate. J. Build.
Eng..

[17] Wang X., Hu Y., Zhu P., Yan X., Chen C., Liu H., Yang L., Xu B., Zong M. (2025). Study on the Effect
of Reduced Graphene Oxide on the Properties of Conductive Recycled
Concrete under Freeze-Thaw Conditions. Constr.
Build. Mater..

[18] Chaturvedy G. K., Kothari P. K., Pandey U. K. (2025). Analyzing
the Effect of Reduced Graphene
Oxide on the Physical, Mechanical, and Long-Term Durability Performance
of Rubberized Concrete. Fullerenes, Nanotubes
Carbon Nanostruct..

[19] Fonseka I., Mohotti D., Wijesooriya K., Lee C.-K., Mendis P. (2025). Evaluating
the Performance of Cement-Reduced Concrete Using Graphene Oxide: Synergistic
Effects on Mechanical Properties. Res. Eng..

[20] Murali M., Alaloul W. S., Mohammed B. S., Musarat M. A., Salaheen M. A., Al-Sabaeei A. M., Isyaka A. (2022). Utilizing Graphene Oxide in Cementitious
Composites: A Systematic Review. Case Stud.
Constr. Mater..

[21] Han S., Hossain M. S., Ha T., Yun K. K. (2022). Graphene-Oxide-Reinforced
Cement Composites Mechanical and Microstructural Characteristics at
Elevated Temperatures. Nanotechnol. Rev..

[22] Dipta, I. A. ; Ng, K. ; Lau, C. K. ; Chadwick, J. ; Johnson, P. Comparative Study of Graphene Oxides from Wyoming Powder River Basin Coal and Commercial Source on Concrete. In American Concrete Institute; https://www.concrete.org/education/freewebsessions/completelisting/coursepreviews.aspx?id=51745561: Philadelphia, PA, USA, 2024.

[23] Reduced graphene oxide - 250MG, 500MG size. https://www.sigmaaldrich.com/US/en/product/aldrich/777684 (accessed Feb 18, 2026).

[24] Reduced Graphene Oxide Powder. Graphenea. https://www.graphenea.com/products/reduced-graphene-oxide-1-gram (accessed Feb 18, 2026).

[25] Graphene Oxide Powder. Graphenea. https://www.graphenea.com/products/graphene-oxide-powder (accessed Feb 18, 2026).

[26] Graphene oxide powder. GREEN GRAPHENE LLC. http://www.green-graphene.com/store/p19/Grapheneoxide_1kg.html (accessed Feb 18, 2026).

[27] Brodie B. C. (1859). On the Atomic Weight of Graphite. Philos. Trans. R. Soc. London.

[28] Hummers W. S., Offeman R. E. (1958). Preparation of Graphitic Oxide. J. Am. Chem. Soc..

[29] Marcano D. C., Kosynkin D. V., Berlin J. M., Sinitskii A., Sun Z., Slesarev A. S., Alemany L. B., Lu W., Tour J. M. (2018). Correction
to Improved Synthesis of Graphene Oxide. ACS
Nano.

[30] Alateah A. H. (2023). Graphene
Concrete: Recent Advances in Production Methods, Performance Properties,
Environmental Impact and Economic Viability. Case Stud. Constr. Mater..

[31] Tamuly J., Bhattacharjya D., Saikia B. K. (2022). Graphene/Graphene
Derivatives from
Coal, Biomass, and Wastes: Synthesis, Energy Applications, and Perspectives. Energy Fuels.

[32] Handiso B., Pääkkönen T., Wilson B. P. (2024). Effect of Pyrolysis
Temperature on the Physical and Chemical Characteristics of Pine Wood
Biochar. Waste Manage. Bull..

[33] Hofmann U., König E. (1937). Untersuchungen
über Graphitoxyd. Z. Anorg. Allg. Chem..

[34] Staudenmaier L. (1898). Verfahren
Zur Darstellung Der Graphitsäure. Ber.
Dtsch. Chem. Ges..

[35] Nazari P., Hamidi A., Golmohammadzadeh R., Rashchi F., Vahidi E. (2024). Upcycling
Spent Graphite in LIBs into Battery-Grade Graphene: Managing the Produced
Waste and Environmental Impacts Analysis. Waste
Manage..

[36] Johnson, P. ; Leandro, A. P. M. Methods for Production of Graphene Oxide. U.S. Patent US20210214231A1, July 15, 2021 https://patents.google.com/patent/US20210214231A1/en?oq=UW+2021%2f0214231 (accessed June 12, 2025).

[37] Yu H., Zhang B., Bulin C., Li R., Xing R. (2016). High-Efficient
Synthesis of Graphene Oxide Based on Improved Hummers Method. Sci. Rep..

[38] Seon
Lee Y., Ryeol Kim N., Ki Park S., Ko Y., Shin Y., Yang B., Yang C.-M. (2024). Effects of High-Temperature Thermal
Reduction on Thermal Conductivity of Reduced Graphene Oxide Polymer
Composites. Appl. Surf. Sci..

[39] ASTM C150/C150M-22 : ASTM Standard Specification for Portland Cement. ASTM International, West Conshohocken, PA, 2022.

[40] ASTM C204–24 . ASTM Standard Test Methods for Fineness of Hydraulic Cement by Air-Permeability Apparatus; ASTM International: West Conshohocken, PA, 2024.

[41] ASTM C29/C29M-23 . ASTM Standard Test Method for Bulk Density (“Unit Weight”) and Voids in Aggregate; ASTM International: West Conshohocken, PA, 2023.

[42] ASTM C127–15 . ASTM Standard Test Method for Relative Density (Specific Gravity) and Absorption of Coarse Aggregate; ASTM International: West Conshohocken, PA, 2015.

[43] ASTM C566–19 . ASTM Standard Test Method for Total Evaporable Moisture Content of Aggregate by Drying; ASTM International: West Conshohocken, PA, 2019.

[44] ASTM C136M-14 : Standard Test Method for Sieve Analysis of Fine and Coarse Aggregates; ASTM International, West Conshohocken, PA, USA.

[45] ASTM C33/C33M-24a . ASTM Standard Specification for Concrete Aggregates; ASTM International: West Conshohocken, PA, 2024.

[46] ASTM C1702–23e1 . ASTM Standard Test Method for Measurement of Heat of Hydration of Hydraulic Cementitious Materials Using Isothermal Conduction Calorimetry; ASTM International: West Conshohocken, PA, 2023.

[47] ACI 211.1–91 . Standard Practice for Selecting Proportions for Normal, Heavyweight, and Mass Concrete 1997.

[48] ASTM C192/C192M-19 . ASTM Standard Practice for Making and Curing Concrete Test Specimens in the Laboratory; ASTM International: West Conshohocken, PA, 2019.

[49] ASTM C143/C143M-20 . ASTM Standard Test Method for Slump of Hydraulic-Cement Concrete; ASTM International: West Conshohocken, PA, 2020.

[50] ASTM C39/C39M-23 . ASTM Standard Test Method for Compressive Strength of Cylindrical Concrete Specimens; ASTM International: West Conshohocken, PA, 2023.

[51] ASTM C78/C78M-22 . ASTM Standard Test Method for Flexural Strength of Concrete (Using Simple Beam with Third-Point Loading); ASTM International: West Conshohocken, PA, 2022.

[52] ASTM C496/C496M-17 . ASTM Standard Test Method for Splitting Tensile Strength of Cylindrical Concrete Specimens; ASTM International: West Conshohocken, PA, 2017.

[53] Standard Test Methods for pH of Soils. https://www.astm.org/d4972-19.html (accessed May 4, 2024).

[54] Jung D.-W., Jeong J.-H., Cha B.-C., Kim J.-B., Kong B.-S., Lee J. K., Oh E.-S. (2011). Effects of Ball-Milled Graphite in
the Synthesis of SnO2/Graphite as an Active Material in Lithium Ion
Batteries. Met. Mater. Int..

[55] Li Z. Q., Lu C. J., Xia Z. P., Zhou Y., Luo Z. (2007). X-Ray Diffraction
Patterns of Graphite and Turbostratic Carbon. Carbon.

[56] Zhou Z., Bouwman W. G., Schut H., Pappas C. (2014). Interpretation
of X-Ray
Diffraction Patterns of (Nuclear) Graphite. Carbon.

[57] Reich S., Thomsen C. (2004). Raman Spectroscopy of Graphite. Philos. Trans. R. Soc., A.

[58] Ferrari A. C., Robertson J. (2000). Interpretation
of Raman Spectra of Disordered and Amorphous
Carbon. Phys. Rev. B.

[59] Vollebregt S., Ishihara R., Tichelaar F. D., Hou Y., Beenakker C. I. M. (2012). Influence
of the Growth Temperature on the First and Second-Order Raman Band
Ratios and Widths of Carbon Nanotubes and Fibers. Carbon.

[60] Pimenta M. A., Dresselhaus G., Dresselhaus M. S., Cançado L. G., Jorio A., Saito R. (2007). Studying Disorder in
Graphite-Based
Systems by Raman Spectroscopy. Phys. Chem. Chem.
Phys..

[61] Taylor, H. F. W. Cement Chemistry; Thomas Telford, 1997.

[62] Zunino F., Scrivener K. (2021). The Reaction
between Metakaolin and Limestone and Its
Effect in Porosity Refinement and Mechanical Properties. Cem. Concr. Res..

[63] Zhang L., Yamauchi K., Li Z., Zhang X., Ma H., Ge S. (2017). Novel Understanding
of Calcium Silicate Hydrate from Dilute Hydration. Cem. Concr. Res..

[64] Qureshi T. S., Panesar D. K. (2019). Impact of Graphene
Oxide and Highly Reduced Graphene
Oxide on Cement Based Composites. Constr. Build.
Mater..

[65] Zhang X., Zhou S., Zhou H., Li D. (2022). The Effect of the Modification
of Graphene Oxide with γ- Aminopropyltriethoxysilane (KH550)
on the Properties and Hydration of Cement. Constr.
Build. Mater..

[66] Zhang H., Gan X., Lu Z., Li L., Lu L. (2025). Polycarboxylate Superplasticizer-Modified
Graphene Oxide: Dispersion and Performance Enhancement in Cement Paste. Nanomaterials.

[67] Djenaoucine L., Picazo A., de la Rubia M. A., Gálvez J. C., Moragues A. (2024). Effect of Graphene Oxide on the Hydration Process and
Macro-Mechanical Properties of Cement. Bol.
Soc. Esp. Ceram. Vidrio.

[68] Gladwin
Alex A., Kedir A., Gebrehiwet Tewele T. (2022). Review on Effects of Graphene Oxide
on Mechanical and Microstructure of Cement-Based Materials. Constr. Build. Mater..

[69] Izadifar M., Dolado J. S., Thissen P., Ayuela A. (2021). Interactions between
Reduced Graphene Oxide with Monomers of (Calcium) Silicate Hydrates:
A First-Principles Study. Nanomaterials.

[70] Kunhi
Mohamed A., Moutzouri P., Berruyer P., Walder B. J., Siramanont J., Harris M., Negroni M., Galmarini S. C., Parker S. C., Scrivener K. L., Emsley L., Bowen P. (2020). The Atomic-Level
Structure of Cementitious Calcium Aluminate Silicate Hydrate. J. Am. Chem. Soc..

[71] Horgnies, M. ; Chen, J. J. ; Bouillon, C. Overview About The Use Of Fourier Transform Infrared Spectroscopy To Study Cementitious Materials. In WIT Transactions on Engineering Sciences; WIT Press, 2013; Vol. 77, pp 251–262 10.2495/MC130221.

[72] Ylmén R., Jäglid U., Steenari B.-M., Panas I. (2009). Early Hydration and
Setting of Portland Cement Monitored by IR, SEM and Vicat Techniques. Cem. Concr. Res..

[73] Xie H., Liu F., Fan Y., Yang H., Chen J., Zhang J., Zuo C. (2013). Workability
and Proportion Design of Pumping Concrete Based on Rheological
Parameters. Constr. Build. Mater..

[74] Sengupta I., Chakraborty S., Talukdar M., Pal S. K., Chakraborty S. (2018). Thermal Reduction
of Graphene Oxide: How Temperature Influences Purity. J. Mater. Res..

[75] Sharma S., C Kothiyal N. (2015). Influence
of Graphene Oxide as Dispersed Phase in Cement
Mortar Matrix in Defining the Crystal Patterns of Cement Hydrates
and Its Effect on Mechanical, Microstructural and Crystallization
Properties. RSC Adv..

[76] Deschner F., Winnefeld F., Lothenbach B., Seufert S., Schwesig P., Dittrich S., Goetz-Neunhoeffer F., Neubauer J. (2012). Hydration of Portland
Cement with High Replacement by Siliceous Fly Ash. Cem. Concr. Res..

[77] Yan Y., Yang S.-Y., Miron G. D., Collings I. E., L’Hôpital E., Skibsted J., Winnefeld F., Scrivener K., Lothenbach B. (2022). Effect of
Alkali Hydroxide on Calcium Silicate Hydrate
(C-S-H). Cem. Concr. Res..

[78] Li X., Korayem A. H., Li C., Liu Y., He H., Sanjayan J. G., Duan W. H. (2016). Incorporation of
Graphene Oxide and
Silica Fume into Cement Paste: A Study of Dispersion and Compressive
Strength. Constr. Build. Mater..

[79] Zhao L., Zhu S., Wu H., Zhang X., Tao Q., Song L., Song Y., Guo X. (2020). Deep Research about the Mechanisms
of Graphene Oxide (GO) Aggregation in Alkaline Cement Pore Solution. Constr. Build. Mater..

[80] Sumra Y., Payam S., Zainah I. (2020). The pH of
Cement-Based Materials:
A Review. J. Wuhan Univ. Technol., Mater. Sci.
Ed..

[81] Yu P., Kirkpatrick R. J., Poe B., McMillan P. F., Cong X. (1999). Structure
of Calcium Silicate Hydrate (C-S-H): Near-, Mid-, and Far-Infrared
Spectroscopy. J. Am. Ceram. Soc..

[82] Guan W., Ji F., Chen Q., Yan P., Pei L. (2013). Synthesis and Enhanced
Phosphate Recovery Property of Porous Calcium Silicate Hydrate Using
Polyethyleneglycol as Pore-Generation Agent. Materials.

[83] Dai J., Wang Y., Luo L., Dong X., Wang Q. (2025). Effects of
Graphene Oxide on Hydration of Cement-Based Materials at Different
Curing Temperatures. J. Build. Eng..

[84] Kong X., Wang R., Zhang T., Sun R., Fu Y. (2022). Effects of
Graphene Oxygen Content on Durability and Microstructure of Cement
Mortar Composites. Constr. Build. Mater..

[85] Zhao L., Guo X., Ge C., Li Q., Guo L., Shu X., Liu J. (2017). Mechanical Behavior
and Toughening Mechanism of Polycarboxylate Superplasticizer
Modified Graphene Oxide Reinforced Cement Composites. Composites, Part B.

[86] Lv S., Ma Y., Qiu C., Sun T., Liu J., Zhou Q. (2013). Effect of
Graphene Oxide Nanosheets of Microstructure and Mechanical Properties
of Cement Composites. Constr. Build. Mater..

[87] Du Y., Yang J., Skariah
Thomas B., Li L., Li H., Mohamed
Shaban W., Tung Chong W. (2020). Influence of Hybrid Graphene Oxide/Carbon
Nanotubes on the Mechanical Properties and Microstructure of Magnesium
Potassium Phosphate Cement Paste. Constr. Build.
Mater..

[88] Horszczaruk E., Mijowska E., Kalenczuk R. J., Aleksandrzak M., Mijowska S. (2015). Nanocomposite of Cement/Graphene Oxide – Impact
on Hydration Kinetics and Young’s Modulus. Constr. Build. Mater..

[89] Lu Z., Hou D., Meng L., Sun G., Lu C., Li Z. (2015). Mechanism
of Cement Paste Reinforced by Graphene Oxide/Carbon Nanotubes Composites
with Enhanced Mechanical Properties. RSC Adv..

[90] Hong X., Lee J. C., Qian B. (2022). Mechanical
Properties and Microstructure
of High-Strength Lightweight Concrete Incorporating Graphene Oxide. Nanomaterials.

